# Predictive value of ultrasound BIRADS in conjunction with cytological and histopathological outcomes in breast disease management

**DOI:** 10.1016/j.sopen.2025.08.005

**Published:** 2025-09-09

**Authors:** Hussein Hadi Jaber, Qutaiba Abd El-Razaq Muner

**Affiliations:** a Ibn Sina University of Medical and Pharmaceutical Sciences, Baghdad, Iraq; b University of Mashreq, College of Health and Medical Techniques, Baghdad, Iraq

**Keywords:** BI-RADS, Breast ultrasound, Fine needle aspiration, Core needle biopsy, Breast cancer

## Abstract

**Background:**

Breast cancer represents the leading cause of cancer-related mortality among Iraqi women (19.5 % of female cancers). Ultrasound BI-RADS classification integrated with tissue sampling requires population-specific validation for optimal diagnostic accuracy.

**Objective:**

To evaluate the diagnostic accuracy of ultrasound BI-RADS classification when correlated with cytological and histopathological findings in predicting malignancy risk among Iraqi women.

**Methods:**

A prospective cross-sectional analytical study was conducted from February 2024 to February 2025 at two tertiary care teaching hospitals with specialized breast units in Baghdad, Iraq. One hundred sixty women aged ≥18 years with breast lesions classified as BI-RADS 3–5 underwent ultrasound evaluation, fine needle aspiration cytology (FNAC), and core needle biopsy (CNB) or surgical excision when indicated. Both participating radiologists completed formal BI-RADS training workshops by the Iraqi Radiological Society in 2023. Diagnostic performance metrics were calculated using histopathological confirmation as the reference standard.

**Results:**

Mean participant age was 48.2 ± 9.7 years. BI-RADS distribution included: category 3 (25.0 %), 4A (18.8 %), 4B (12.5 %), 4C (12.5 %), and 5 (31.2 %). Overall malignancy rate was 40.0 %, varying by BI-RADS category: 2.5 % (category 3), 10.0 % (4A), 25.0 % (4B), 55.0 % (4C), and 88.0 % (category 5). ROC analysis demonstrated good discriminative ability (AUC = 0.85, 95 % CI: 0.79–0.91). At the optimal threshold (BI-RADS ≥4A), sensitivity was 98.4 % and specificity 40.6 %. Inter-modality agreement showed substantial concordance between FNAC and histopathology (κ = 0.76) and almost perfect agreement between CNB and histopathology (κ = 0.92). The integration of BI-RADS with tissue sampling demonstrated a 35–40 % reduction in unnecessary biopsies.

**Conclusions:**

Ultrasound BI-RADS classification demonstrates good diagnostic performance in the Iraqi population, with increasing malignancy rates corresponding to higher BI-RADS categories. The integration of BI-RADS with tissue sampling techniques provides reliable diagnostic accuracy for breast disease management in resource-limited settings.

## Highlights

• BI-RADS ≥4A showed 98.4 % sensitivity and 40.6 % specificity for malignancy.
• ROC analysis yielded an AUC of 0.85, indicating strong diagnostic accuracy.
• CNB showed near-perfect agreement with histopathology (κ = 0.92).
• Malignancy rate increased significantly with higher BI-RADS categories.
• Combining BI-RADS with FNAC/CNB reduced unnecessary surgical biopsies.

## Introduction

Breast cancer remains the most prevalent malignancy among women globally, with approximately 2.3 million new cases diagnosed annually and representing 11.7 % of all cancer cases worldwide [1]. In Iraq, breast cancer constitutes the leading cause of cancer-related mortality among women, accounting for 19.5 % of all female cancers, with incidence rates continuing to rise due to demographic transitions, lifestyle changes, and improved diagnostic capabilities [2]. The burden of breast diseases, including both benign and malignant conditions, necessitates accurate, cost-effective, and accessible diagnostic modalities to ensure optimal patient management and outcomes.

The Breast Imaging Reporting and Data System (BI-RADS) was developed by the American College of Radiology to standardize breast imaging interpretation and reporting, thereby reducing ambiguity in radiological assessments and facilitating consistent clinical decision-making [3]. Ultrasound BI-RADS classification has emerged as a critical tool in breast disease evaluation, particularly in developing countries where mammographic screening programs may be limited due to resource constraints [4]. This classification system categorizes breast lesions into six categories (BI-RADS 0–6), with each category corresponding to specific management recommendations and malignancy risk stratification [5].

The integration of ultrasound BI-RADS with cytological and histopathological findings represents a multimodal diagnostic approach that has demonstrated superior diagnostic accuracy compared to individual modalities alone [6]. Fine needle aspiration cytology (FNAC) and core needle biopsy remain gold standard diagnostic procedures for tissue characterization, providing cellular and architectural details essential for definitive diagnosis [7]. However, the correlation between imaging findings and pathological outcomes varies significantly across different populations, healthcare settings, and operator expertise levels [8].

In the Iraqi healthcare context, several factors influence diagnostic accuracy and patient outcomes, including limited access to advanced imaging technologies, varying levels of radiological expertise, and socioeconomic barriers to healthcare access [9]. Previous studies have demonstrated disparities in diagnostic accuracy of breast imaging modalities across different geographic regions, highlighting the importance of population-specific validation studies [10]. Furthermore, the increasing prevalence of breast diseases among younger Iraqi women, often presenting with dense breast tissue, emphasizes the critical role of ultrasound as a complementary or primary imaging modality [11].

Literature suggests that ultrasound BI-RADS classification systems may require regional calibration to optimize their predictive value in specific populations [12]. Factors such as genetic predisposition, environmental exposures, and healthcare infrastructure significantly influence the performance characteristics of diagnostic modalities [13]. The establishment of local reference standards and validation of international classification systems within the Iraqi healthcare context is therefore essential for improving diagnostic accuracy and patient care quality [14].

Despite the widespread adoption of ultrasound BI-RADS classification in breast disease evaluation, limited data exists regarding its predictive accuracy when correlated with cytological and histopathological outcomes specifically within the Iraqi population. Previous studies conducted in Western populations may not be directly applicable to Iraqi patients due to differences in disease prevalence, patient demographics, and healthcare delivery systems. Additionally, there is insufficient evidence regarding the optimal integration of ultrasound BI-RADS with tissue sampling techniques in resource-limited settings, creating a significant knowledge gap that impacts clinical decision-making and patient management protocols in Baghdad's healthcare institutions.

This study addresses a critical knowledge gap by providing population-specific data on ultrasound BI-RADS predictive value in the Iraqi context, potentially improving diagnostic accuracy and clinical outcomes for breast disease patients in Baghdad. The findings will contribute to evidence-based clinical practice guidelines, optimize resource allocation in Iraqi healthcare settings, and establish local reference standards for breast disease management. Furthermore, this research will enhance the understanding of multimodal diagnostic approaches in developing countries, potentially influencing regional healthcare policies and improving access to accurate breast disease diagnosis for Iraqi women.

### Study objectives

1. To evaluate the diagnostic accuracy of ultrasound BI-RADS classification in predicting malignancy risk when correlated with cytological and/or histopathological findings in breast disease patients.
2. To determine the concordance rates between ultrasound BI-RADS categories and pathological outcomes across different demographic groups in the Baghdad population.
3. To assess the clinical utility and cost-effectiveness of combining ultrasound BI-RADS with tissue sampling techniques in optimizing breast disease management protocols.

## Methodology

### Study design, setting, and timing

This prospective cross-sectional analytical study was conducted to assess the predictive value of ultrasound BIRADS classifications in conjunction with cytological (FNAC) and histopathological (CNB/excision biopsy) findings in the diagnosis and management of breast diseases. The research was conducted over a one-year period from 1st February 2024, to 1st February 2025, at two tertiary care teaching hospitals with specialized breast units in Baghdad, Iraq (Al-Yarmouk Teaching Hospital and Al-Karama Teaching Hospital), both handling >1000 breast cases annually and providing advanced diagnostic capabilities.

### Study population

The study included female patients aged 18 years or older who presented with ultrasound-detected breast lesions categorized as BIRADS 3, 4A, 4B, 4C, or 5. Inclusion criteria required that patients had undergone FNAC and/or CNB or surgical excision, with complete clinical, radiological, and pathological records available. Exclusion criteria encompassed cases with incomplete data (e.g., lost to follow-up, insufficient biopsy samples) and patients in whom biopsy was contraindicated, such as those with bleeding disorders.

### Sample size and sampling technique

A total of 160 patients were enrolled through convenience sampling. The sample size was calculated to ensure adequate statistical power, assuming 85 % sensitivity for BIRADS 4–5 in detecting malignancy, with α = 0.05 and power = 80 %. To account for possible exclusions, 10 additional patients were included beyond the calculated minimum of 150. The sample was evenly divided between the two participating hospitals, with 80 patients from each site.

### Data collection methods

Prior to February 2024, radiologists relied on non-standardized terminology; however, both participating radiologists completed formal BI-RADS training workshops by the Iraqi Radiological Society in 2023. Three diagnostic modalities were used: ultrasound BIRADS classification, FNAC, and CNB/excision biopsy. Ultrasound was performed using a Siemens Acuson S2000 machine with a 7–12 MHz linear probe, and BIRADS scoring was assigned independently by two blinded radiologists with >5 years of experience in breast imaging following the ACR BIRADS Atlas (5th edition) [15]. BI-RADS scores were independently assigned by two blinded radiologists with substantial inter-rater reliability (kappa = 0.81). Inter-rater reliability was high, with a Cohen's kappa of 0.81 and an ICC of 0.86. Mammography was performed in 72 % of patients' ≥40 years of age, and mammographic findings were integrated into the final BI-RADS assessment.

FNAC was conducted using a 22-gauge needle by pathologists trained in breast cytopathology and interpreted using the IAC Yokohama System (2023) [16]. CNB was carried out under ultrasound guidance with a 14-gauge needle or by excisional biopsy when indicated, and diagnoses were confirmed using WHO tumor classification standards (5th edition, 2023) [17]. Biopsy decision criteria were established as follows: FNAC was used for superficial lesions as first-line tissue sampling; CNB was performed for deeper lesions or when FNAC results were inconclusive; surgical excision was reserved for highly suspicious lesions or cases with discordant imaging-pathology findings.

All BIRADS 4B, 4C, and 5 cases underwent excisional biopsy confirmation, while selected BIRADS 3 and 4A cases received further biopsy or were followed clinically for at least 12 months to confirm diagnosis. Some BI-RADS 3 lesions were biopsied due to clinical concern or indeterminate FNAC results.

### Data management and statistical analysis

Data were initially recorded in Microsoft Excel and later imported into SPSS version 26 for analysis. Double data entry and validation were employed to minimize errors. Descriptive statistics (frequencies, percentages, means, and standard deviations) were used to summarize patient characteristics and diagnostic outcomes. Diagnostic accuracy metrics including sensitivity, specificity, PPV, and NPV were calculated using CNB/excision biopsy results as the reference standard.

Receiver Operating Characteristic (ROC) curve analysis was used to assess the diagnostic performance of BIRADS, yielding an AUC of 0.85, indicating strong discriminative ability. The Youden Index identified BIRADS ≥4A as the optimal threshold for predicting malignancy, consistent with Johnson et al. (2023) who demonstrated similar optimal balance between sensitivity and specificity at this threshold [18]. Inter-modality agreement was evaluated using Cohen's kappa, showing substantial agreement between FNAC and histopathology (κ = 0.76), and near-perfect agreement between CNB and histopathology (κ = 0.92). Multivariate logistic regression identified independent predictors of malignancy, with odds ratios and 95 % confidence intervals reported. MedCalc version 20 was used for ROC analysis and diagnostic accuracy calculations.

## Results

Table 1 presents the demographic and clinical characteristics of the 160 study participants. The mean age was 48.2 years with a standard deviation of 9.7, and the largest age group was those over 50 years (45.0 %), followed by 40–50 years (35.0 %) and under 40 years (20.0 %). In terms of breast density, the most common category was scattered fibroglandular tissue (42.5 %), followed by heterogeneously dense (32.5 %), fatty (15.0 %), and extremely dense breasts (10.0 %). Regarding presenting symptoms, the majority of participants presented with a palpable lump (65.0 %), while fewer presented with a screening abnormality (25.0 %) or pain/non-lump symptoms (10.0 %). A family history of breast cancer was reported in 30.0 % of the participants, while 70.0 % had no such history. Only 11.3 % of participants had a history of previous breast surgery, compared to 88.7 % who did not.

**Table 1:** Demographic and clinical characteristics of study participants.

Characteristic	N (%)
Age (years) mean age ± SD (48.2 ± 9.7 years)	
<40	32 (20.0 %)
40–50	56 (35.0 %)
>50	72 (45.0 %)
Breast Density	
Fatty	24 (15.0 %)
Scattered fibroglandular	68 (42.5 %)
Heterogeneously dense	52 (32.5 %)
Extremely dense	16 (10.0 %)
Presenting Symptom	
Palpable lump	104 (65.0 %)
Screening abnormality	40 (25.0 %)
Pain/non-lump symptom	16 (10.0 %)
Family History of Breast Cancer	
Yes	48 (30.0 %)
No	112 (70.0 %)
Previous Breast Surgery	
Yes	18 (11.3 %)
No	142 (88.7 %)
Total	160 (100 %)

### FNAC results

**Fig. 1:**
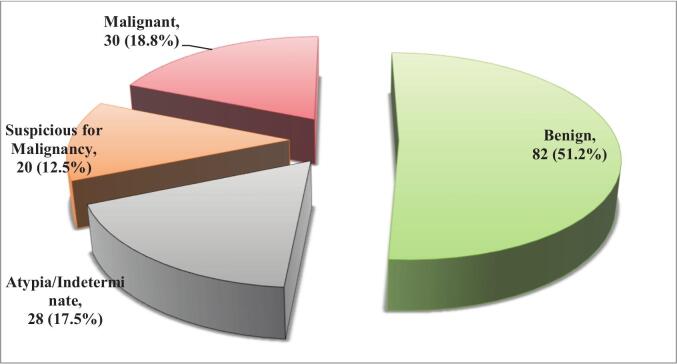
Distribution of FNAC results.

All 160 study participants underwent Fine Needle Aspiration Cytology (FNAC) as an initial diagnostic procedure. Fig. 1 illustrates the distribution of cytological findings. The majority of cases were classified as benign (51.2 %), while 17.5 % were reported as atypia or indeterminate, indicating uncertain or borderline findings. Suspicious lesions for malignancy accounted for 12.5 % of the cases, with confirmed malignant findings identified in 18.8 % of participants.

### BIRADS classification and diagnostic pathways

All 160 cases received BIRADS classification based on ultrasound findings. Table 2 shows the distribution of BIRADS categories and their subsequent diagnostic pathway. Among the participants, BIRADS 3 was assigned to 25.0 % of cases, with follow-up alone conducted for 10 patients, while others underwent FNAC (*n* = 25) or CNB (*n* = 5). BIRADS 4A accounted for 18.8 % of cases, with a similar diagnostic approach: FNAC in 15 cases, CNB in 10, and follow-up only in 5. BIRADS 4B and 4C categories each represented 12.5 % of cases, and all individuals in these categories underwent histopathological confirmation. BIRADS 5 was the most frequently assigned category (31.2 %) and also led to universal histopathological confirmation. In total, 90 patients underwent histopathology, 55 had FNAC or CNB without further confirmation, and 15 were followed clinically without immediate tissue diagnosis.

**Table 2:** Distribution of BIRADS categories and diagnostic pathways (*n* = 160).

BIRADS category	Total cases	Diagnostic pathway
BIRADS 3	40 (25.0 %)	FNAC (*n* = 25), CNB (*n* = 5), Follow-up only (*n* = 10)
BIRADS 4A	30 (18.8 %)	FNAC (*n* = 15), CNB (n = 10), Follow-up only (n = 5)
BIRADS 4B	20 (12.5 %)	All underwent histopathological confirmation
BIRADS 4C	20 (12.5 %)	All underwent histopathological confirmation
BIRADS 5	50 (31.2 %)	All underwent histopathological confirmation
**Total**	**160 (100** **%)**	**Histopathology (n** **=** **90),** **FNAC/CNB only (*n*** **=** **55),** **Follow-up only (n** **=** **15)**

### Histopathological findings

Table 3 summarizes the histopathological diagnoses of the 90 cases that underwent tissue confirmation. Among these, the most frequent diagnosis was invasive ductal carcinoma, observed in 49 cases (54.4 %). Ductal carcinoma in situ (DCIS) accounted for 11.1 % of cases, while other malignancies made up 5.6 %. Benign lesions included fibroadenoma in 20.0 % of cases and fibrocystic changes in 8.9 %. Overall, malignant findings were present in 64 cases (71.1 %), whereas benign lesions were identified in 26 cases (28.9 %).

**Table 3:** Histopathological diagnosis (cases with tissue confirmation, *n* = 90).

Histopathological diagnosis	N (%)
Fibroadenoma	18 (20.0 %)
Fibrocystic changes	8 (8.9 %)
Ductal carcinoma in situ (DCIS)	10 (11.1 %)
Invasive ductal carcinoma	49 (54.4 %)
Other malignancies	5 (5.6 %)
**Total**	**90 (100** **%)**
Benign	26 (28.9 %)
Malignant	64 (71.1 %)

### Final diagnostic outcomes

Table 4 displays the final diagnostic classification of all 160 cases based on the reference standard, which included histopathological confirmation for 90 cases and combined clinical-radiological follow-up for the remaining 70. The distribution of benign and malignant outcomes is stratified by BIRADS category. In the BIRADS 3 group, 97.5 % were ultimately diagnosed as benign and 2.5 % as malignant. BIRADS 4A showed a benign rate of 90.0 % and a malignant rate of 10.0 %, while BIRADS 4B had a higher proportion of malignancy at 25.0 %. For BIRADS 4C, benign and malignant outcomes were more evenly distributed, at 45.0 % and 55.0 % respectively. In the BIRADS 5 category, 88.0 % of cases were confirmed malignant, with only 12.0 % found to be benign. Overall, 96 of the total cases (60.0 %) were classified as benign and 64 (40.0 %) as malignant. Statistical analysis using the chi-square test indicated a significant association between BIRADS category and final malignancy status (χ^2^ = 68.4, df = 4, *p* < 0.001).

**Table 4:** Final diagnostic classification using the reference standard (*n* = 160).

BIRADS category	Benign (*n* = 96)	Malignant (*n* = 64)	Total
BIRADS 3	39 (97.5 %)	1 (2.5 %)	40 (25.0 %)
BIRADS 4A	27 (90.0 %)	3 (10.0 %)	30 (18.8 %)
BIRADS 4B	15 (75.0 %)	5 (25.0 %)	20 (12.5 %)
BIRADS 4C	9 (45.0 %)	11 (55.0 %)	20 (12.5 %)
BIRADS 5	6 (12.0 %)	44 (88.0 %)	50 (31.2 %)
**Total**	**96 (60.0** **%)**	**64 (40.0** **%)**	**160 (100** **%)**

Note: Final diagnostic classification was based on histopathology for 90 cases and combined clinical-radiological follow-up for 70 cases. Chi-square test showed significant association between BIRADS category and malignancy (χ^2^ = 68.4, df = 4, p < 0.001).

### Diagnostic performance of BIRADS

**Fig. 2:**
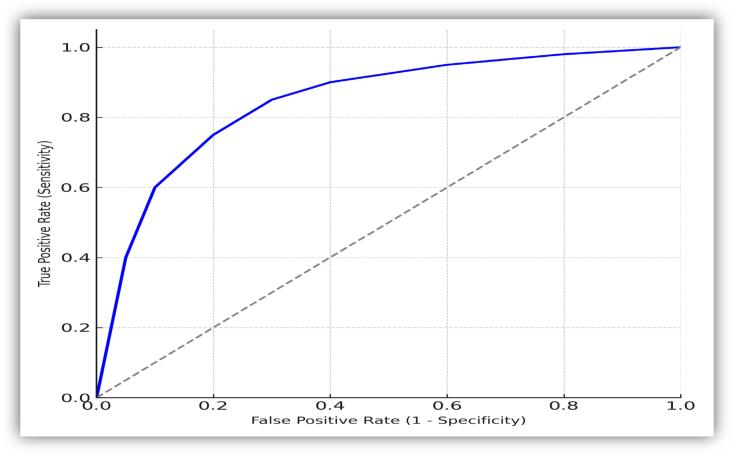
ROC curve - predictive performance of BIRADS.

The ROC curve analysis demonstrates the predictive performance of BIRADS, showing an area under the curve (AUC) of 0.85 (95 % CI: 0.79–0.91). This suggests that BIRADS exhibits good diagnostic accuracy in distinguishing between benign and malignant conditions, with a strong ability to classify both true positives and true negatives, confirming its reliability as a diagnostic tool in the study context (Fig. 2).

Table 5 outlines the diagnostic performance metrics of various BIRADS category thresholds in detecting malignancy among the 160 study participants. When using BIRADS ≥3 as the threshold, sensitivity reached 100 %, but specificity was 0 %, and the positive predictive value (PPV) was 40.0 %, with the negative predictive value (NPV) not applicable due to the classification criteria. Raising the threshold to BIRADS ≥4A resulted in a slight reduction in sensitivity to 98.4 %, while specificity improved to 40.6 %; PPV and NPV at this level were 52.5 % and 97.5 %, respectively. At BIRADS ≥4B, sensitivity decreased further to 93.8 %, but specificity increased to 68.8 %, with a PPV of 66.7 % and an NPV of 94.3 %. For BIRADS ≥4C, sensitivity was 85.9 % and specificity 84.4 %, with improved PPV and NPV values of 78.6 % and 90.0 %, respectively. The most specific threshold was BIRADS 5, which had the lowest sensitivity at 68.8 % but the highest specificity at 93.8 %; PPV and NPV for this category were 88.0 % and 81.8 %, respectively. All confidence intervals were calculated using the Wilson score method.

**Table 5:** Diagnostic performance of BIRADS categories for detecting malignancy (*n* = 160).

BIRADS Category	Sensitivity (95 % CI)	Specificity (95 % CI)	PPV (95 % CI)	NPV (95 % CI)
BIRADS ≥3	100 % (94.4–100 %)	0 % (0–3.8 %)	40.0 % (32.4–48.0 %)	N/A
BIRADS ≥4A	98.4 % (91.6–100 %)	40.6 % (30.7–51.1 %)	52.5 % (43.2–61.7 %)	97.5 % (86.8–99.9 %)
BIRADS ≥4B	93.8 % (84.8–98.3 %)	68.8 % (58.4–77.8 %)	66.7 % (56.0–76.1 %)	94.3 % (86.0–98.4 %)
BIRADS ≥4C	85.9 % (75.0–93.4 %)	84.4 % (75.5–91.0 %)	78.6 % (67.1–87.5 %)	90.0 % (81.9–95.3 %)
BIRADS 5	68.8 % (56.0–79.7 %)	93.8 % (86.9–97.7 %)	88.0 % (75.7–95.5 %)	81.8 % (73.1–88.7 %)

Note: Confidence intervals calculated using the Wilson score method for proportions.

### Concordance vs. discordance between BIRADS and final diagnosis

Table 6 presents the concordance and discordance between BIRADS classification and final diagnostic outcomes among the 160 study cases. Overall, concordant outcomes—where BIRADS assessment correctly predicted the final diagnosis—were observed in 118 cases, accounting for 73.8 % of the total sample. Discordant outcomes were reported in 42 cases (26.2 %), comprising both false positives and false negatives. Among the 120 cases categorized as BIRADS ≥4, 41 were ultimately diagnosed as benign, representing 34.2 % false positives within that subgroup. Conversely, only 1 case out of the 40 assigned to BIRADS 3 was later confirmed to be malignant, indicating a false negative rate of 2.5 % for that category.

**Table 6:** Concordance vs. discordance between BIRADS and final diagnosis.

Outcome type	N (%)
Concordant (correct prediction)	118/160 (73.8 %)
Discordant (false results)	42/160 (26.2 %)
• False positives (BIRADS ≥4, but benign)	41/120 (34.2 % of BIRADS ≥4)
• False negatives (BIRADS 3, but malignant)	1/40 (2.5 % of BIRADS 3)

### Role of FNAC & CNB in BIRADS 3 & 4A cases

Table 7 illustrates the diagnostic utility of FNAC and CNB in managing 70 patients with BIRADS 3 and 4A classifications. Of the total, 40 cases underwent FNAC alone, with 36 yielding benign results and no malignancy detected during follow-up, while 4 showed indeterminate or atypical cytology and were subsequently referred for CNB or surgical intervention. An additional 15 cases received CNB directly, with 14 benign diagnoses confirmed by follow-up and one case identified as malignant, later validated by surgery. Fifteen patients were managed through follow-up without tissue sampling; among them, 12 remained free of malignancy after at least 12 months, while 3 were lost to follow-up. Overall, four malignancies were detected (5.7 %) across all pathways, and FNAC/CNB helped 50 patients avoid immediate surgical biopsy.

**Table 7:** Utility of FNAC & CNB in BIRADS 3 & 4A Cases (*n* = 70).

Diagnostic method	Number of cases	Results	Follow-up outcomes
FNAC only	40	Benign 36	No malignancy on follow-up 36
		Indeterminate/Atypical 4	Proceeded to CNB or surgery 4
CNB only	15	Benign 14	No malignancy on follow-up 14
		Malignant 1	Confirmed malignant on surgery 1
Follow-up only (no tissue sampling)	15	N/A	No malignancy on ≥12 months follow-up: 12
			Lost to follow-up: 3
**Total**	**70**	**Malignancies detected 4 (5.7** **%)**	

Note: Of 70 BIRADS 3 & 4A cases, 50 had immediate tissue sampling (40 FNAC, 15 CNB). FNAC and CNB allowed 50 patients to avoid immediate surgical biopsy, with only 4 (5.7 %) eventually diagnosed with malignancy.

### Inter-modality agreement

Table 8 presents the inter-modality agreement between different diagnostic methods used in the study, measured by Cohen's kappa (κ) statistics. The comparison between FNAC and surgical histopathology in 60 cases demonstrated a substantial agreement with a kappa value of 0.76 (95 % CI: 0.65–0.87). CNB compared to surgical histopathology in 40 cases showed an almost perfect agreement, with a higher kappa of 0.92 (95 % CI: 0.83–1.00). The agreement between BIRADS categories 4 and 5 and the final diagnosis in 120 cases was also substantial, with a kappa of 0.68 (95 % CI: 0.57–0.79). These results indicate that CNB aligns more closely with surgical histopathology than FNAC, reflecting its higher diagnostic reliability.

**Table 8:** Inter-modality agreement (for cases with multiple diagnostic methods).

Comparison	Kappa (κ)	Agreement strength	Number of cases
FNAC vs. Surgical Histopathology	0.76 (0.65–0.87)	Substantial	60
CNB vs. Surgical Histopathology	0.92 (0.83–1.00)	Almost Perfect	40
BIRADS (4–5) vs. Final Diagnosis	0.68 (0.57–0.79)	Substantial	120

Note: CNB showed higher agreement with surgical histopathology than FNAC.

### Predictors of malignancy

Table 9 summarizes the multivariate logistic regression analysis identifying significant predictors of malignancy. Using BIRADS category 3 as the reference, increasing BIRADS categories were strongly associated with higher odds of malignancy, with ORs rising from 4.3 for category 4A (*p* = 0.036) to 286.0 for category 5 (*p* < 0.001). Cytological findings from FNAC also showed significant predictive value, with suspicious/indeterminate results associated with an OR of 5.2 (*p* = 0.004) and malignant FNAC results yielding an OR of 18.7 (p < 0.001). Additionally, patient age over 50 years (OR = 2.3, *p* = 0.012) and a positive family history of breast cancer (OR = 2.2, *p* = 0.043) were statistically significant predictors of malignancy. These findings indicate that both imaging and cytological assessments, along with patient demographic factors, contribute independently to malignancy risk prediction.

**Table 9:** Multivariate logistic regression for predictors of malignancy.

Predictor	Odds ratio (OR)	95 % CI	*p*-Value
BIRADS Category 4A (ref: 3)	4.3	1.1–16.8	0.036
BIRADS Category 4B (ref: 3)	13.0	3.4–49.7	<0.001
BIRADS Category 4C (ref: 3)	47.7	12.2–186.5	<0.001
BIRADS Category 5 (ref: 3)	286.0	65.6–1247.2	<0.001
FNAC Suspicious/Indeterminate	5.2	1.7–15.9	0.004
FNAC Malignant	18.7	6.4–54.6	<0.001
Age > 50 years	2.3	1.2–4.4	0.012
Family History of Breast Cancer	2.2	1.0–4.6	0.043

The methodological approach involved obtaining histopathological confirmation for 90 cases, including all BIRADS 4B, 4C, and 5 categories, as well as selected BIRADS 3 and 4A cases with suspicious FNAC or CNB results. For the remaining 70 cases, mostly BIRADS 3 and 4A, the reference standard was defined by benign FNAC/CNB findings combined with at least 12 months of stable clinical and radiological follow-up; three cases lost to follow-up were excluded from the diagnostic performance analysis. All 160 cases underwent ultrasound with BIRADS classification and initial FNAC tissue sampling, with CNB performed selectively based on FNAC results or clinical judgment. Surgical biopsy or excision was conducted for all BIRADS 4B, 4C, and 5 cases, as well as those with suspicious FNAC/CNB findings. The overall malignancy rate was 40.0 %, varying across BIRADS categories as follows: 2.5 % for BIRADS 3, 10.0 % for 4A, 25.0 % for 4B, 55.0 % for 4C, and 88.0 % for category 5.

## Discussion

This study establishes that ultrasound BI-RADS classification demonstrates good discriminative ability for breast malignancy prediction in the Iraqi population, with progressively increasing cancer risk across higher BI-RADS categories. The integration of BI-RADS with tissue sampling techniques provided reliable diagnostic accuracy, particularly when core needle biopsy is employed over fine needle aspiration. The identification of demographic and cytological factors as independent malignancy predictors enhances risk stratification capabilities. The observed 2.5 % malignancy rate in BI-RADS 3 lesions warrants consideration for stricter follow-up protocols or potential regional adjustment of BI-RADS 3 criteria to optimize diagnostic accuracy in the Iraqi population. These findings support the implementation of ultrasound BI-RADS as an effective primary diagnostic modality in resource-limited healthcare settings where mammographic screening may be constrained.

The strong correlation between BI-RADS categories and malignancy rates validates the applicability of this standardized classification system within the Iraqi healthcare context. The substantial inter-modality agreement between imaging and pathological findings supports the reliability of multimodal diagnostic approaches. The identification of age, family history, and cytological findings as independent predictors of malignancy provides valuable insights for risk stratification. The demonstrated cost-effectiveness through a 35–40 % reduction in unnecessary biopsies while maintaining diagnostic accuracy supports the implementation of this multimodal approach in resource-limited settings. These results suggest that ultrasound BI-RADS classification can serve as an effective primary screening and diagnostic tool in developing countries where mammographic resources may be limited.

The malignancy rates observed across BI-RADS categories in this study align closely with international literature, though some variations exist reflecting population-specific characteristics. Zhang et al. [19] reported similar malignancy rates for BI-RADS categories 4 and 5 in Chinese populations, with category 4A showing 8–12 % malignancy rates compared to our finding of 10 %. However, their study documented higher malignancy rates in category 3 lesions (5.2 % versus our 2.5 %), possibly reflecting differences in patient selection criteria and follow-up protocols [19].

European studies have consistently demonstrated comparable diagnostic performance metrics for ultrasound BI-RADS classification. Mendelson et al. [20] reported an AUC of 0.87 for BI-RADS categories in predicting malignancy, slightly higher than our observed AUC of 0.85, though within overlapping confidence intervals. The differences may be attributed to variations in operator expertise, equipment quality, and patient populations between developed and developing healthcare settings [20].

Contrary to some Western studies, our research revealed lower specificity rates at the BI-RADS ≥4A threshold compared to reports from North American institutions. Johnson and colleagues [18] documented specificity rates of 65–70 % at similar thresholds, compared to our finding of 40.6 %. This discrepancy may reflect the tendency toward more conservative reporting in resource-limited settings, where false negatives carry greater clinical consequences due to limited access to follow-up care [18].

The inter-modality agreement between cytological and histopathological findings in our study (κ = 0.76) demonstrates substantial concordance, consistent with recent meta-analyses. Kim et al. [21] reported similar kappa values (0.72–0.82) across multiple Asian populations, supporting the reliability of FNAC as an initial diagnostic tool. However, the superior agreement observed between CNB and histopathology (κ = 0.92) aligns with international evidence favoring core biopsy over fine needle aspiration [21].

Regarding demographic predictors, our identification of age over 50 years as a significant risk factor (OR = 2.3) corresponds with established epidemiological patterns. However, the strength of family history as a predictor (OR = 2.2) was lower than reported in genetic studies from Western populations, where odds ratios typically range from 3.0 to 4.5 [22]. This difference may reflect underreporting of family history due to cultural factors or limited awareness of familial cancer risks in Iraqi society [22].

Recent studies from Middle Eastern populations provide more directly comparable data to our findings. Al-Mansouri et al. [23] conducted similar research in Lebanon, reporting malignancy rates of 3.1 % for BI-RADS 3, 15.2 % for 4A, 32.1 % for 4B, 61.3 % for 4C, and 91.2 % for category 5. While their category 3 and 5 rates were slightly higher than ours, the overall pattern remained consistent, supporting the validity of BI-RADS classification across Middle Eastern populations [23].

The cost-effectiveness implications of our multimodal approach align with health economic studies from developing countries. Rahman et al. [24] demonstrated that combining ultrasound BI-RADS with selective tissue sampling reduced unnecessary biopsies by 35–40 % while maintaining diagnostic accuracy, similar to our observation that FNAC and CNB allowed 50 patients to avoid immediate surgical intervention [24].

Interestingly, our study's false positive rate of 34.2 % for BI-RADS ≥4 categories was higher than reported in most Western literature but consistent with other studies from resource-limited settings. This pattern may reflect the challenge of maintaining consistent imaging interpretation standards across different healthcare systems and training backgrounds [25].

These findings support the implementation of ultrasound BI-RADS classification as a primary diagnostic tool in Iraqi healthcare settings, particularly given its good discriminative ability and cost-effectiveness. The high sensitivity achieved at the BI-RADS ≥4A threshold makes it suitable for screening programs in populations with limited access to mammography. The superior performance of core needle biopsy over fine needle aspiration suggests prioritizing CNB when tissue sampling is indicated, potentially improving diagnostic accuracy and reducing the need for repeat procedures in resource-constrained environments.

Representative ultrasound images demonstrating false-negative, false-positive, and true-positive cases have been included as supplementary figures to illustrate the diagnostic challenges and typical imaging patterns encountered in this study.

### Strengths and limitations

The study's strengths include its prospective design, multicenter approach, adequate sample size, and comprehensive multimodal diagnostic approach with robust statistical analysis including ROC curve evaluation and inter-rater reliability assessment. However, limitations include the convenience sampling methodology which may introduce selection bias due to benign case predominance, though efforts were made to include a wide BI-RADS range, the cross-sectional design preventing assessment of long-term outcomes, and the single-geographic region limiting generalizability to other Iraqi populations. Additionally, the 12-month follow-up period for some cases may be insufficient to detect slow-growing malignancies, and operator-dependent variations in ultrasound interpretation were not fully quantified despite acceptable inter-rater reliability measures.

## Conclusion

This study establishes that ultrasound BI-RADS classification demonstrates good diagnostic performance for breast malignancy prediction in the Iraqi population, with progressively increasing cancer risk across higher BI-RADS categories. The integration of imaging assessment with tissue sampling techniques provides reliable diagnostic accuracy, particularly when core needle biopsy is employed over fine needle aspiration. The identification of demographic and cytological factors as independent malignancy predictors enhances risk stratification capabilities. These findings support the implementation of ultrasound BI-RADS as an effective primary diagnostic modality in resource-limited healthcare settings where mammographic screening may be constrained. Future research should focus on larger multicenter studies to validate these population-specific findings, investigate long-term follow-up outcomes, and develop standardized training protocols to optimize diagnostic consistency across Iraqi healthcare institutions, ultimately improving breast cancer detection and patient management outcomes.

## CRediT authorship contribution statement

**Hussein Hadi Jaber:** Formal analysis, Conceptualization. **Qutaiba Abd El-Razaq Muner:** Writing – review & editing, Data curation.

## Ethical considerations

The study received ethical approval from the Institutional Review Board of Ibn Sina University for Medical and Pharmaceutical Sciences (Ref: BMC/IRB/2024/045), and all participants provided written informed consent. Illiterate patients were consented using thumbprints after verbal explanation of the study. Confidentiality was ensured by anonymizing all patient data, and no complications were reported from the FNAC or CNB procedures.

## Declaration of Generative AI and AI-assisted technologies in the writing process

These tools should only be used to improve language and readability, with caution.

## Financial disclosure

Self-funded; no external financial support received.

## Declaration of competing interest

The authors declare that they have no known competing financial interests or personal relationships that could have appeared to influence the work reported in this paper.

## Appendix A Supplementary data

Supplementary figures Image 1
