# Supplementary material for: Predictive value of ultrasound BIRADS in conjunction with cytological and histopathological outcomes in breast disease management
Source: Surg Open Sci. 2025 Sep 9;27:171–8. doi: 10.1016/j.sopen.2025.08.005 (PMC12540029; doi:10.1016/j.sopen.2025.08.005)
Supplement: Supplementary file 1 — Supplementary figures [file mmc1.docx]

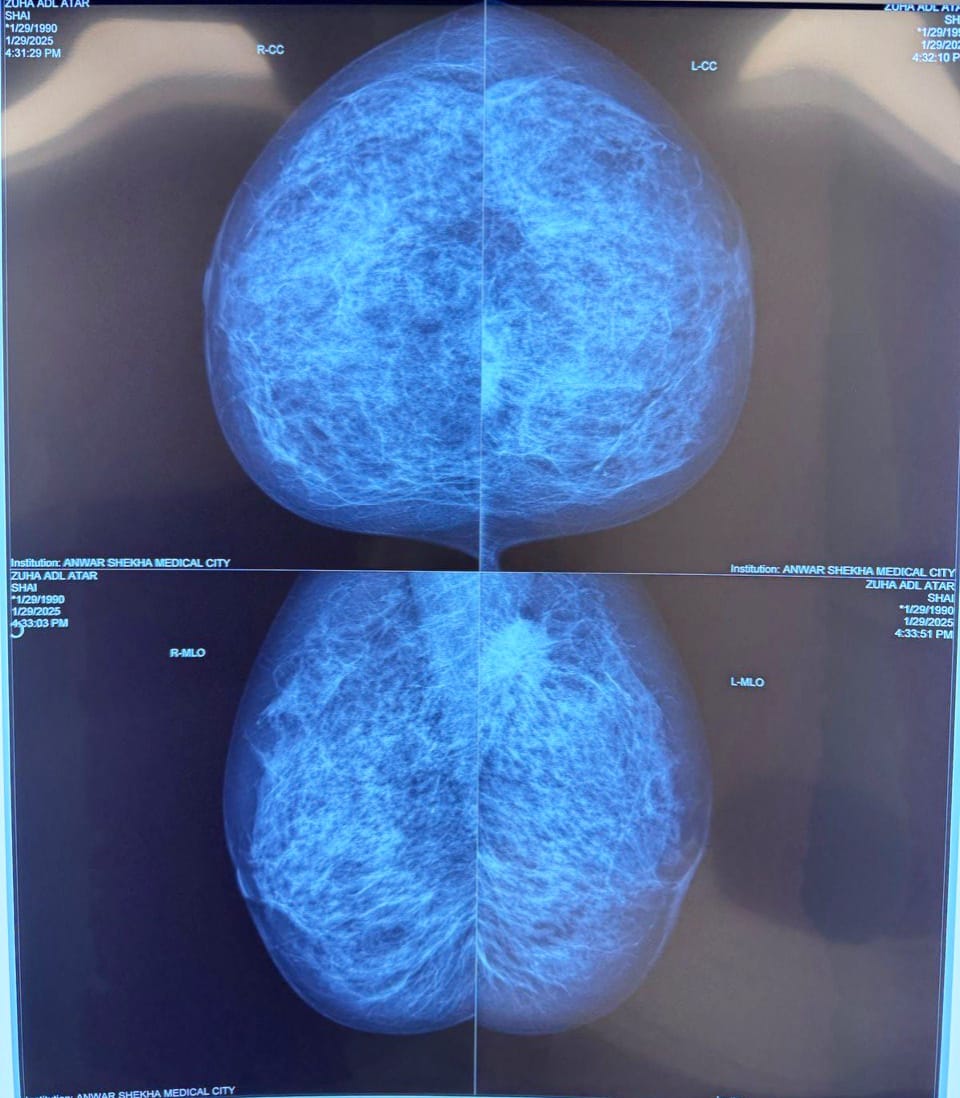
***Figure S1:*** *Mammography Image Illustrating a False-Negative Finding*


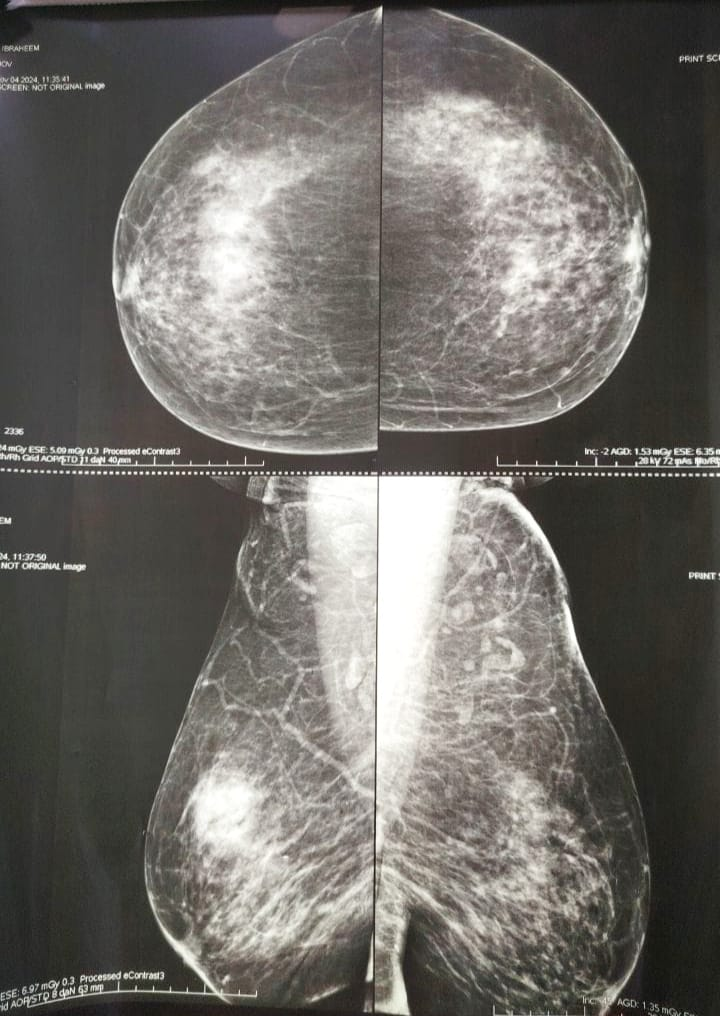


***Figure S2:*** *Mammography Image Depicting a False-Positive Diagnosis*


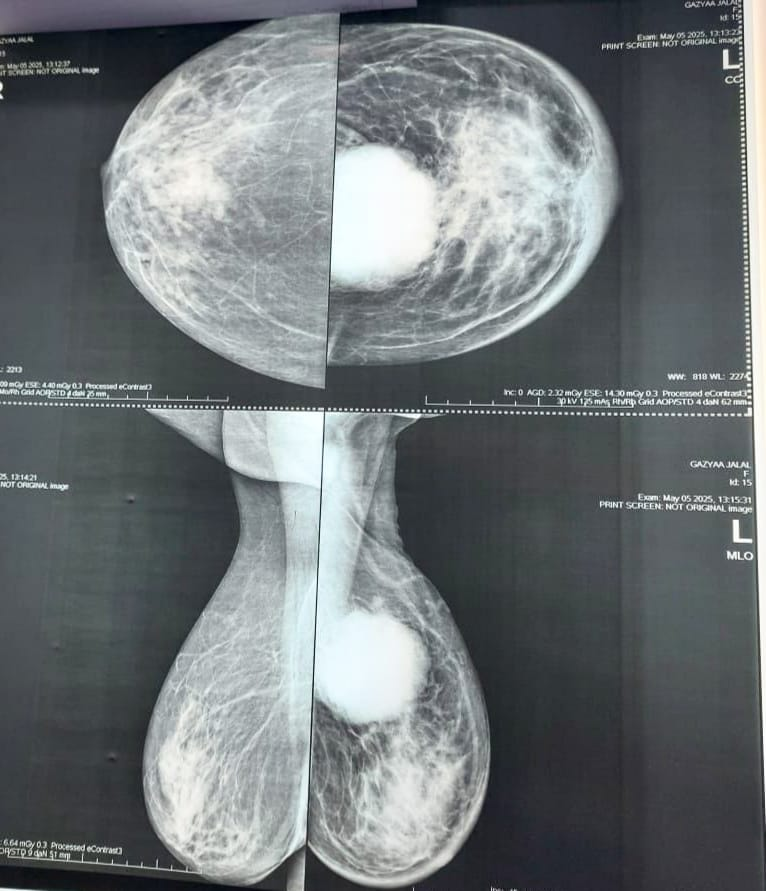


***Figure S3:*** *Mammography Image Demonstrating a True-Positive Detection*
